# Supplementary material for: Distributed neural representations of conditioned threat in the human brain
Source: Nat Commun. 2024 Mar 12;15:2231. doi: 10.1038/s41467-024-46508-0 (PMC10933283; doi:10.1038/s41467-024-46508-0)
Supplement: Supplementary file 1 — Supplementary Information [file 41467_2024_46508_MOESM1_ESM.pdf]

Supplementary Information

Distributed neural representations of conditioned threat in the human brain

Zhenfu Wen, Edward F. Pace-Schott, Sara W. Lazar, Jörgen Rosén, Fredrik Åhs, Elizabeth A. Phelps, Joseph E. LeDoux & Mohammed R. Milad

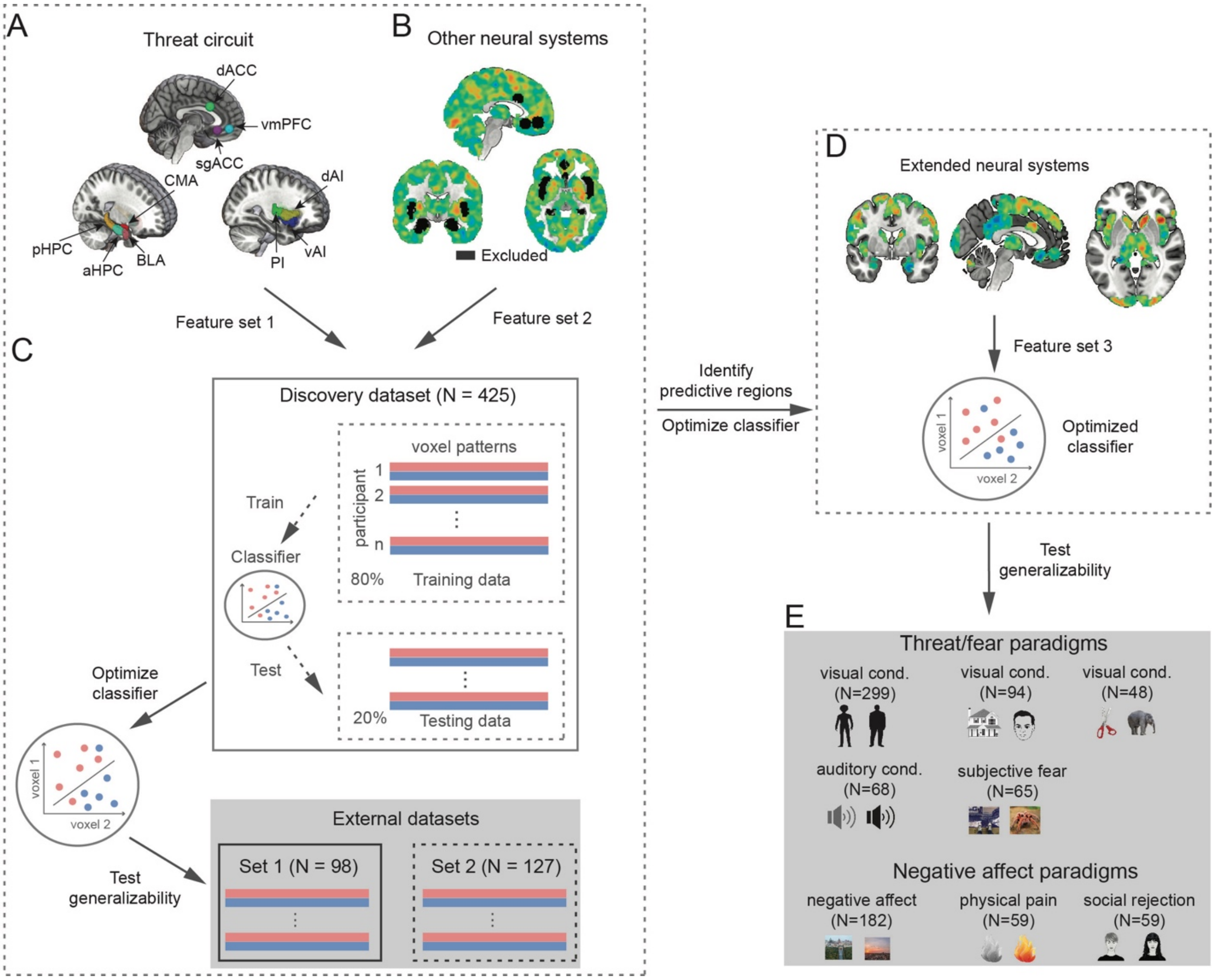

Supplementary Fig. 1. Overview of the analyses. A. The ‘threat circuit’. **B.** The other neural systems with voxels from the ‘threat circuit’ excluded. **C.** The cross-validation and the external validation procedures. **D.** The updated ‘threat detection and flexible responding circuit’. **E.** The external datasets used for the validation.

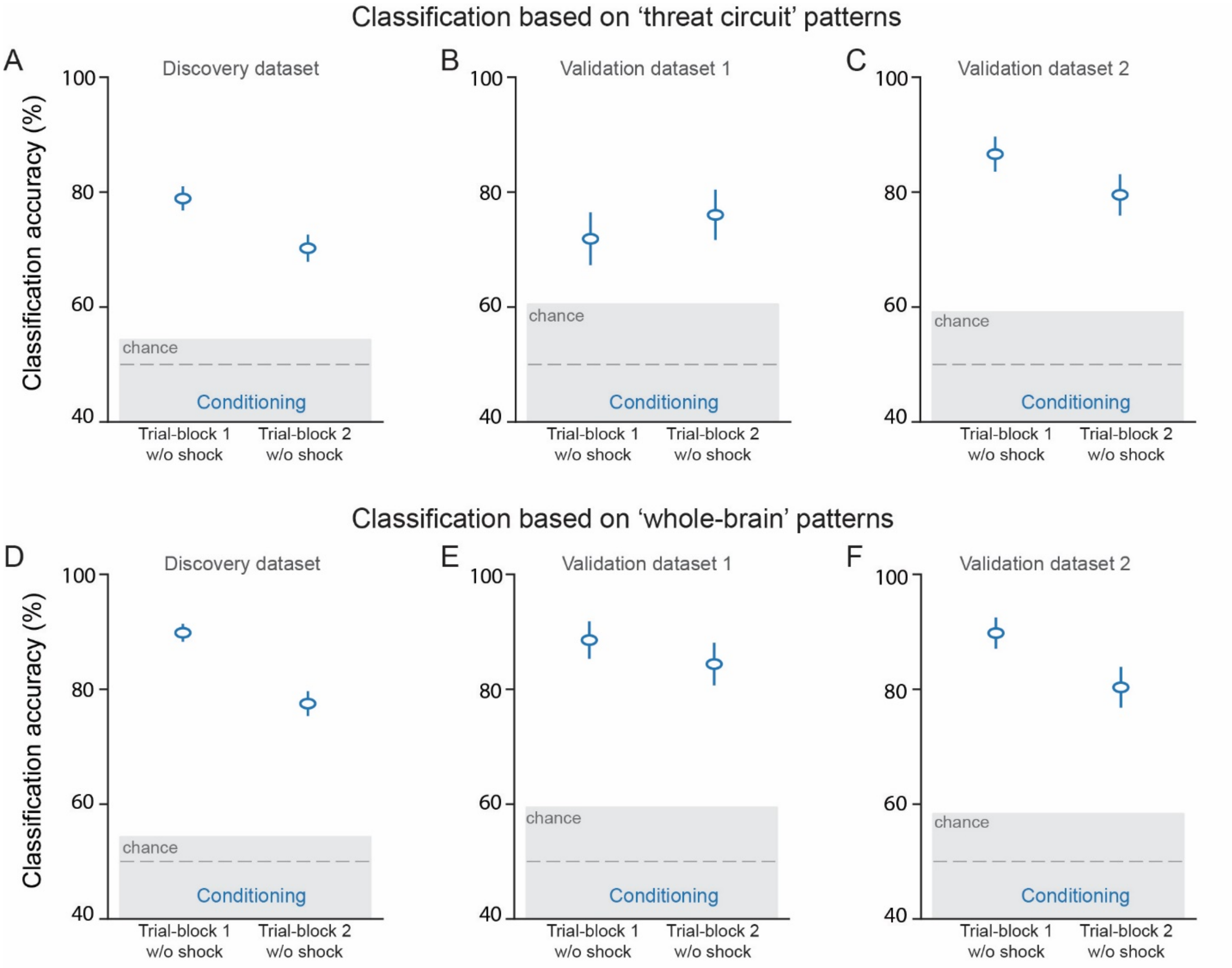

Supplementary Fig. 2. Classification in the conditioning phase by only including unreinforced CS+ trials. A-C, Classification performance based on 'threat circuit' patterns on the discovery dataset (*n* = 425), validation dataset 1 (*n* = 98), and validation dataset 2 (*n* = 127). **D-F**, Classification performance based on 'whole-brain' patterns on the discovery dataset, validation dataset 1, and validation dataset 2. Error bars are SEM. The unreinforced CS+ trials were divided into two trial-blocks (3 CS+ trials in each trial-block). Activation maps for unreinforced CS+ trials and corresponding CS- trials within each trial-block were estimated and used for classification analysis.

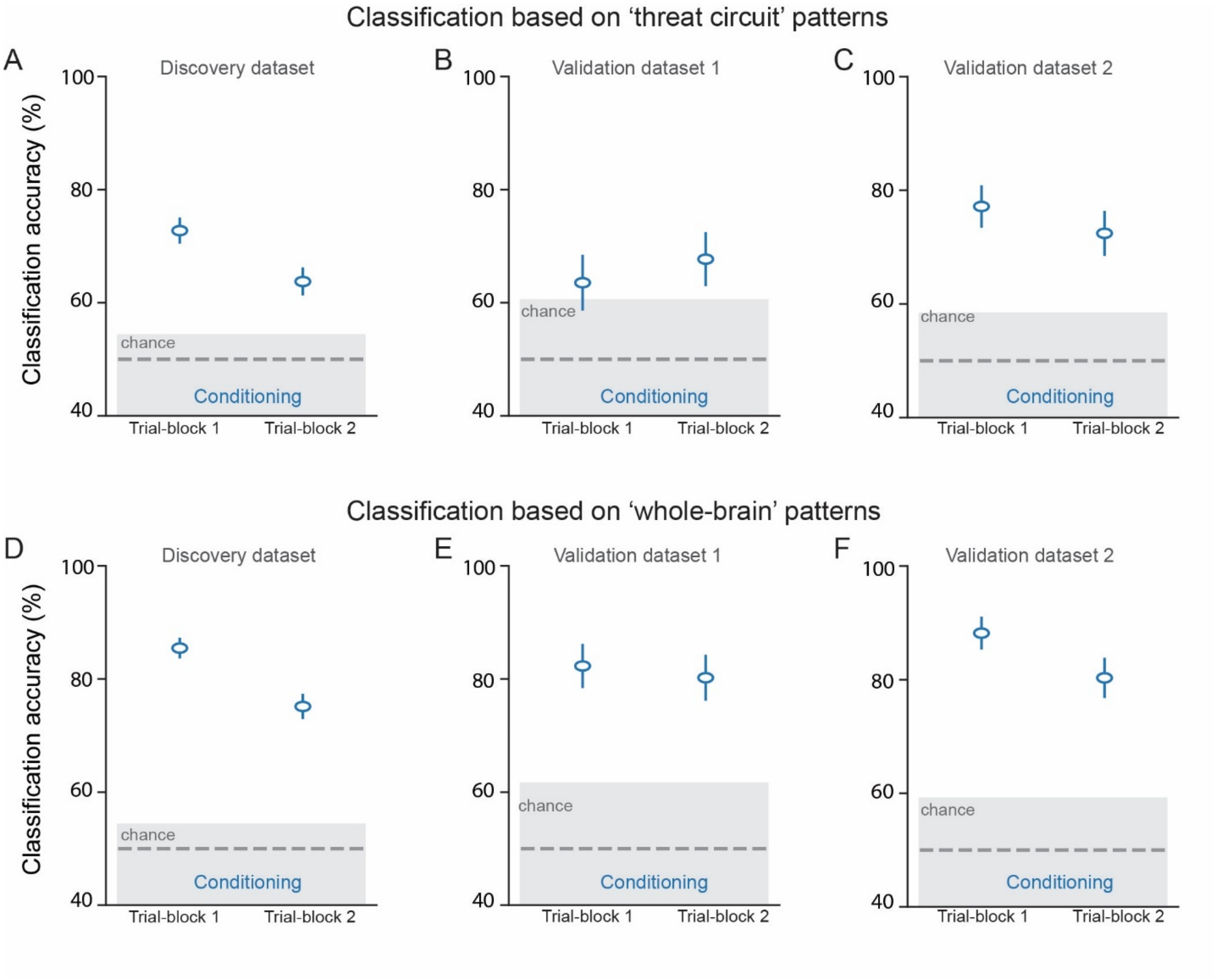

Supplementary Fig. 3. Classification in the conditioning phase by only including trials not confounded by shock. A-C, Classification performance based on 'threat circuit' patterns on the discovery dataset (*n* = 425), validation dataset 1 (*n* = 98), and validation dataset 2 (*n* = 127). **D-F**, Classification performance based on 'whole-brain' patterns on the discovery dataset, validation dataset 1, and validation dataset 2. Error bars are SEM. To fully exclude the impact of shock on the classification, we exclusively included trials that are not followed by a shock, and not preceded by a shock (so that preceding shock signal is not picked up by the classifier) for the classification. This resulted in 2 CS+ trials and 2 CS- trials in each trial-block for the classification analysis.

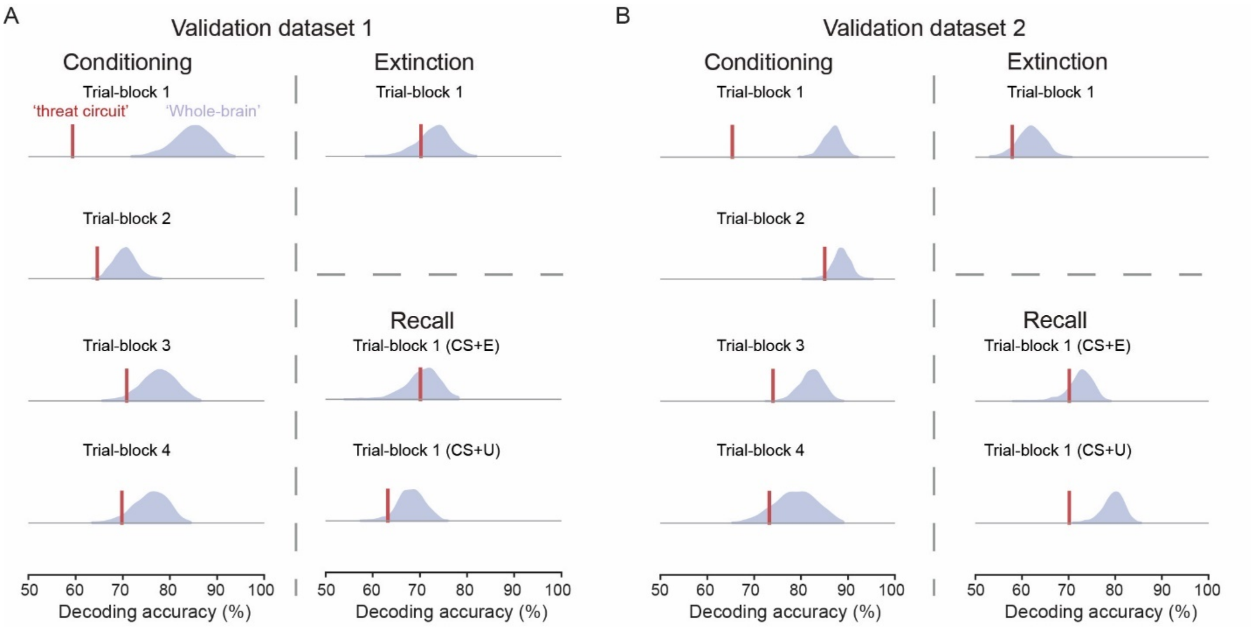

Supplementary Fig. 4. Improved classification performance using 'whole-brain' patterns over the 'threat circuit' can not be fully explained by increased voxel number. A. Generalizations on external dataset 1 (*n* = 98). **B.** Generalizations on external dataset 2 (*n* = 127). The red vertical lines indicate the decoding accuracies obtained using 'threat circuit' patterns. The blue distributions indicate the decoding accuracies obtained using voxel patterns beyond the 'threat circuit' but with the number of voxels matched with the 'threat circuit'. We randomly sampled the same number of voxels as the 'threat circuit' from 'whole-brain' patterns (1000 times) for model training, and applied the trained models to validation datasets 1 and 2.

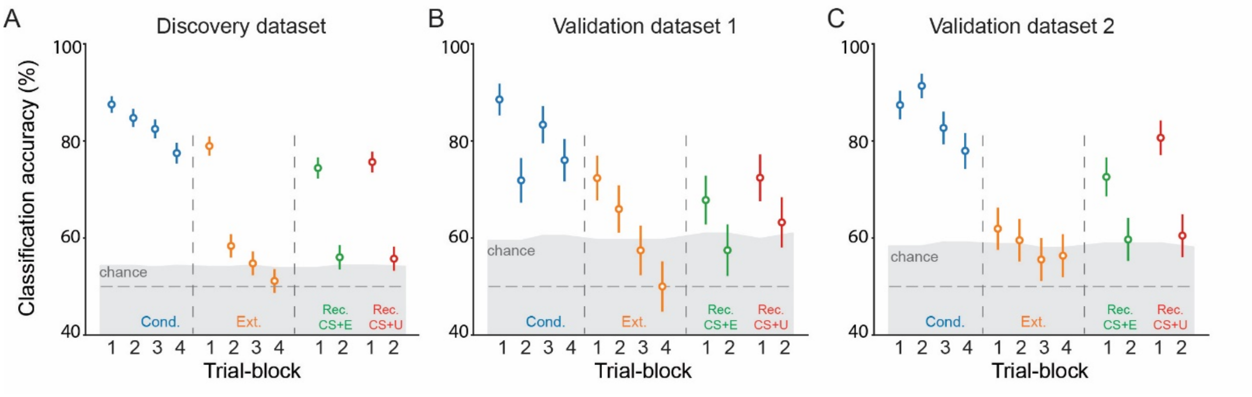

Supplementary Fig. 5. Classification based on all gray matter patterns. A. Cross-validation accuracies on the discovery dataset (*n* = 425). **B**. Generalization accuracies on the validation dataset 1 (*n* = 98). **C**. Generalization accuracies on the validation dataset 2 (*n* = 127). Error bars are SEM. Activation patterns of all gray matter voxels (including the 'threat circuit' voxels) were used for the classification.

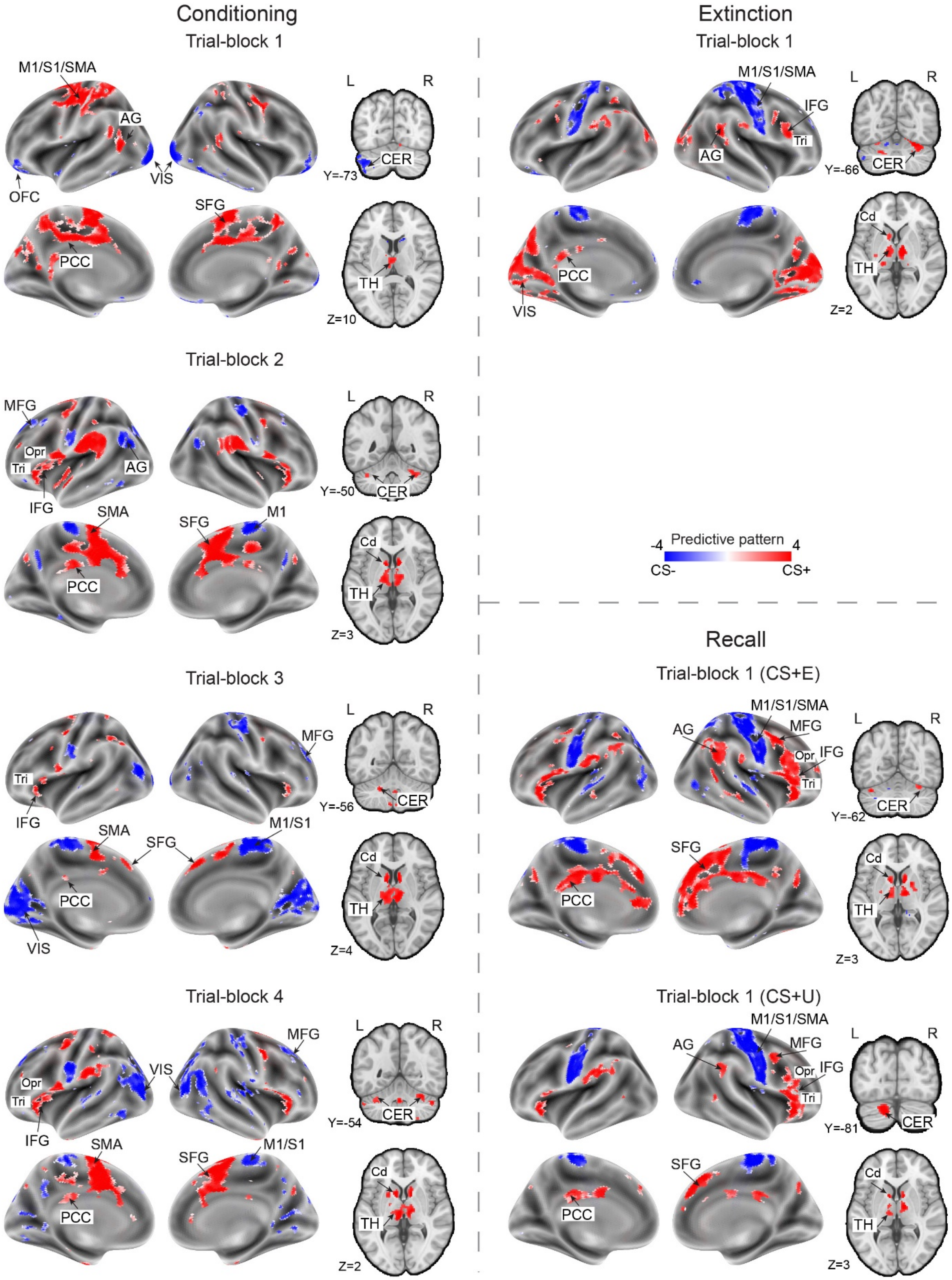

Conditioning
Trial-block 1
M1/S1/SMA
AG
VIS
OFC
L
R
CER
Y=-73
SFG
PCC
TH
Z=10
Trial-block 2
MFG
Opr
Tri
AG
IFG
Y=-50
CER
SMA
SFG
M1
PCC
Cd
TH
Z=3
Trial-block 3
MFG
Tri
IFG
Y=-56
CER
SMA
SFG
M1/S1
PCC
VIS
Cd
TH
Z=4
Trial-block 4
MFG
Opr
Tri
VIS
IFG
Y=-54
CER
SMA
SFG
M1/S1
PCC
Cd
TH
Z=2
Extinction
Trial-block 1
M1/S1/SMA
IFG
Tri
AG
Y=-66
CER
PCC
VIS
Cd
TH
Z=2
-4 Predictive pattern 4
CS-
CS+
Recall
Trial-block 1 (CS+E)
M1/S1/SMA
AG
MFG
Opr
IFG
Tri
CER
Y=-62
SFG
PCC
Cd
TH
Z=3
Trial-block 1 (CS+U)
M1/S1/SMA
AG
MFG
Opr
IFG
Tri
Y=-81
CER
SFG
PCC
Cd
TH
Z=3

Supplementary Fig. 6. Predictive patterns of voxels across the brain. A voxel with red/blue color indicates that this voxel is more activated to CS+/CS-. Permutation tests were conducted to assess the voxel contributions to the classification. Only voxels that significantly contributed to the classification (p < 0.05, FDR-corrected, two-sided) are shown. Regions included in the ‘extended circuit’ were marked.

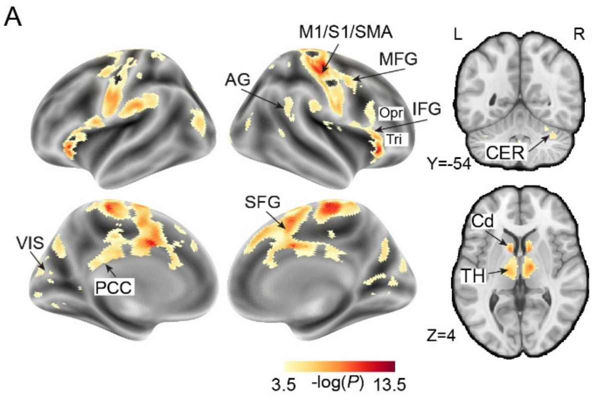

Supplementary Fig. 7. Voxels with the largest contributions to the classification across trial-blocks. A. The top 10% voxels with the largest contributions. **B.** The top 20% voxels with the largest contributions. Regions included in the 'extended circuit' were marked.

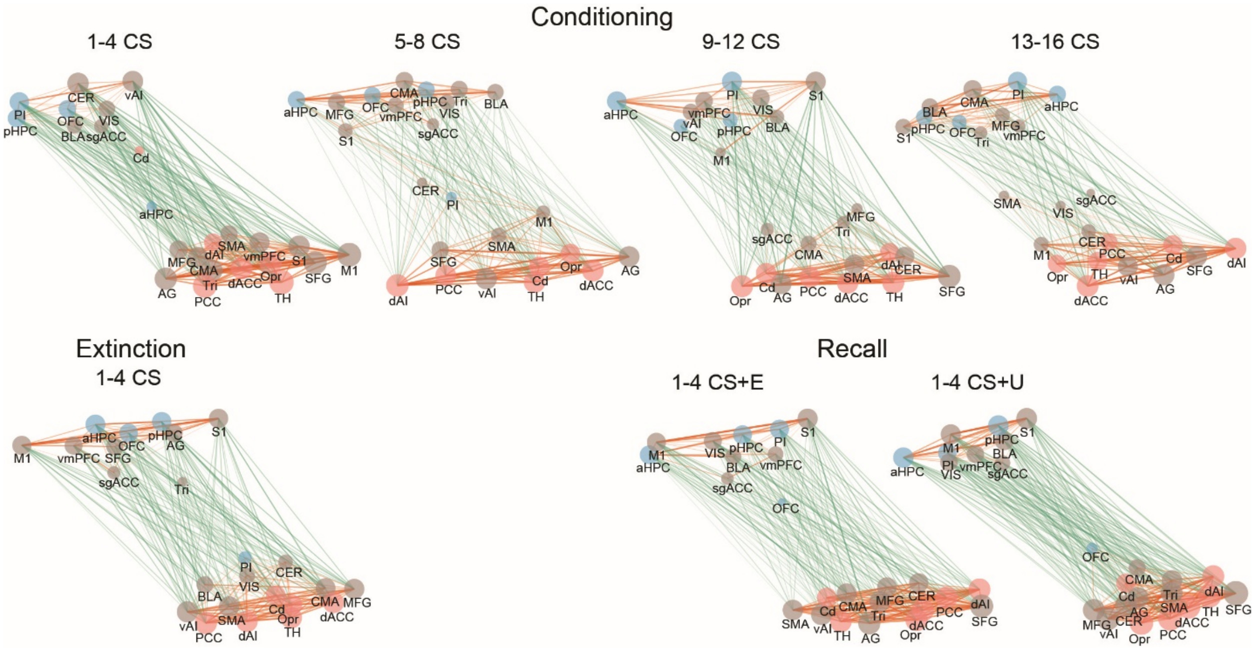

Supplementary Fig. 8. The representational similarity analysis. Each circle represents a brain region. The size of each circle is proportional to the mean connection between this region and other regions. The circles were colored based on their preference for the threat and safe cues: red-colored regions consistently code the threat cue (CS+) across experimental phases; blue-colored regions consistently code the safe cue (CS-) across experimental phases; Gray-colored regions dynamically code CS+ or CS- depending on the experimental phase. The edge between the two regions represents the similarity of their contributions to the decoding. The red edge means a similar contribution, green edge means the opposite contribution.

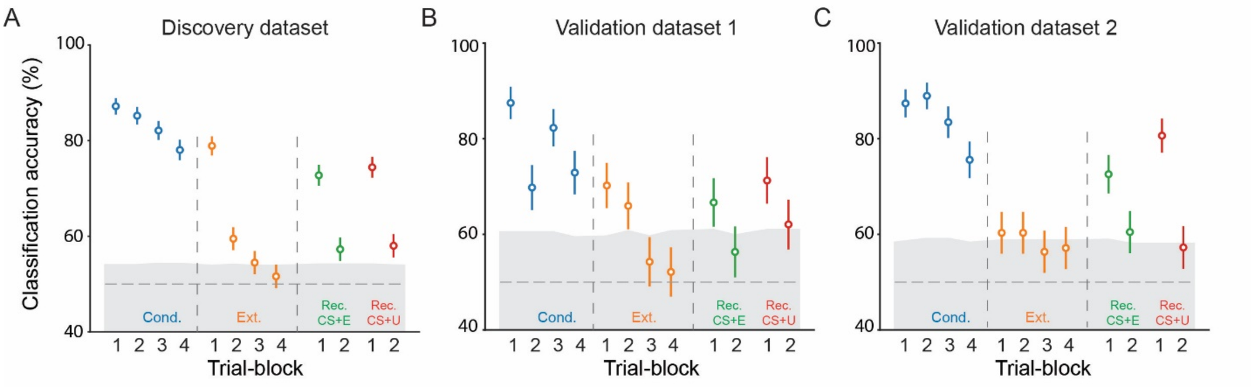

Supplementary Fig. 9. Classification with more voxels from the 'threat circuit' excluded. A. Cross-validation accuracies on the discovery dataset (n = 425). **B**. Generalization accuracies on the validation dataset 1 (n = 98). **C**. Generalization accuracies on the validation dataset 2 (n = 127). Error bars are SEM. Instead of defining vmPFC, dACC, sgACC using 8 mm spheres, we used a more inclusive mask to define these regions. The mask includes the anterior cingulate cortex, paracingulate cortex, and a larger part of vmPFC. The vmPFC was defined by including frontal pole, frontal medial cortex, subcallosal cortex, paracingulate gyrus, anterior cingulate gyrus and frontal orbital cortex (areas dorsal to Z = 0 mm and lateral to X = ± 12 mm are excluded). All regions were defined based on the Harvard-Oxford atlas. We excluded voxels from this inclusive mask and the other nodes of the 'threat circuit', and then conducted the 'whole-brain' classification analysis.

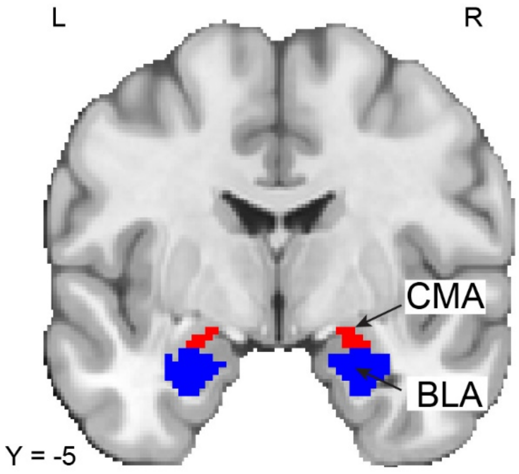

Supplementary Fig. 10. The map of amygdala masks. BLA: Basolateral amygdala; CMA: Centromedial amygdala.

Supplementary Table 1. Basic information of discovery dataset and validation datasets 1-2.

Dataset	Training or testing	Demographic information	Key scanning information	Paradigm description	Original publications
Discovery	Training	Sample size: 31 (45% female); Age (SD): 32.1 (11.5); Diagnosis: 16 PTSD, 15 TENC.	Setting 1: Siemens 3T MRI scanner, 8-channel head-coil. Functional data TR: 3.0 s, slice number: 45, voxel size: 3 × 3 × 3 mm.	Threat conditioning and extinction paradigm, using colors as CS (duration: 6 s), 62.5% reinforcement rate. Threat conditioning: 16 CS+, 16 CS-; Extinction learning: 16 CS+, 16 CS-; Extinction memory recall: 8 CS+E, 8 CS+U, 16 CS-.	Milad et al. 2009[1].
Discovery	Training	Sample size: 34 (100%); Age: 23.2 (2.6); Diagnosis: 34 HC.	Same as setting 1.	Same as above.	Zeidan et al. 2011[2].
Discovery	Training	Sample size: 57 (41%); Age: 27.9 (8.2); Diagnosis: 57 HC.	Same as setting 1.	Same as above.	Milad et al. 2007[3], Milad et al. 2013[4], Holt et al. 2012[5].
Discovery	Training	Sample size: 65 (54%); Age: 34.0(13.2); Diagnosis: 21 HC, 24 PTSD, 20 TENC.	Setting 2; Siemens 3T MRI scanner, 32-channel head-coil. Functional data TR: 2.56 s, slice number: 48, voxel size: 3 × 3 × 3 mm.	Same as above.	Marin et al. 2016[6].
Discovery	Training	Sample size: 114 (61%); Age: 30.5 (12.0); Diagnosis: 21 HC, 93 ANX.	Same as setting 2.	Same as above.	Marin et al. 2020[7].
Discovery	Training	Sample size: 124 (76.6%); Age: 38.6 (17.5); Diagnosis: 125 HC	Same as setting 2.	Same as above.	Unpublished.
Validation dataset 1	Testing	Sample size: 98 (64.3%); Age: 32.0 (8.4); Diagnosis: 98 HC.	Setting 3. Siemens 3T MRI scanner, 32-channel head-coil. Functional data TR: 3.0 s, slice number: 48, voxel size: 2.5 × 2.5 × 2.5 mm.	Same as above.	Sevinc et al. 2019[8].
Validation dataset 2	Testing	Sample size: 127 (68.5%); Age: 24.6 (5.0); Diagnosis: 63 PTSD, 64 TENC.	Same as setting 2.	Same as above.	Seo et al. 2021[9].

PTSD: Post-traumatic stress disorder; TENC: Trauma-exposed non-PTSD control; ANX: Anxiety disorder; HC: Healthy control; SD: Standard deviation.

Supplementary Table 2. Classification performance on discovery dataset.

Phase	Threat circuit' patterns			Whole-brain' patterns		
	Accuracy	P-value	95% CI	Accuracy	P-value	95% CI
Conditioning TB1	**70.5%*** **	p<0.001	[65.9%, 75.1%]	**88.6%*** **	p<0.001	[85.4%, 91.8%]
Conditioning TB2	**74.0%*** **	p<0.001	[69.3%, 78.3%]	**85.7%*** **	p<0.001	[82.0%, 89.4%]
Conditioning TB3	**65.5%*** **	p<0.001	[60.6%, 70.4%]	**81.7%*** **	p<0.001	[77.8%, 85.7%]
Conditioning TB4	**68.7%*** **	p<0.001	[64.0%, 73.3%]	**77.5%*** **	p<0.001	[73.3%, 82.0%]
Extinction TB1	**62.4%*** **	p<0.001	[57.6%, 66.9%]	**80.2%*** **	p<0.001	[76.2%, 84.0%]
Extinction TB2	53.4%	0.077	[48.6%, 58.3%]	**60.5%*** **	p<0.001	[55.5%, 65.0%]
Extinction TB3	52.9%	0.12	[48.3%, 57.9%]	**55.5%* **	0.022	[50.7%, 60.2%]
Extinction TB4	51.6%	0.26	[46.9%, 56.4%]	49.3%	0.64	[44.8%, 54.3%]
Recall TB1 (CS+E vs. CS-)	**68.0%*** **	p<0.001	[63.2%, 72.8%]	**74.6%*** **	p<0.001	[70.4%, 78.8%]
Recall TB1 (CS+U vs. CS-)	**67.4%*** **	p<0.001	[62.5%, 71.9%]	**72.6%*** **	p<0.001	[68.1%, 77.0%]
Recall TB2 (CS+E vs. CS-)	**56.9%** **	0.001	[52.1%, 62.0%]	**56.8%** **	0.002	[52.1%, 62.0%]
Recall TB2 (CS+U vs. CS-)	52.2%	0.19	[47.4%, 57.3%]	**56.5%** **	0.002	[51.6%, 61.5%]

Accuracies significantly higher than change level (permutation test, p<0.05) were bolded. Since 1000 shuffles were used for the permutation tests, the resolution of p-values was 0.001 (1/1000). We reported p<0.001 in cases where the real accuracy was higher than all accuracies in the null distribution. TB: Trial-block; CI: Confidence interval; ***: p<0.001; **: p<0.01; *: p<0.05.

Supplementary Table 3. Classification performance on validation datasets 1-2.

Phase	'Threat circuit' patterns				'Whole-brain' patterns			
	Validation dataset 1		Validation dataset 2		Validation dataset 1		Validation dataset 2	
	Accuracy	P-value	Accuracy	P-value	Accuracy	P-value	Accuracy	P-value
Conditioning TB1	59.40%	0.082	**65.4%****	0.007	**80.2%*****	1.86E-9	**87.4%*****	1.12E-17
Conditioning TB2	**64.6%****	0.006	**85.0%*****	2.65E-16	**70.0%*****	1.32E-4	**90.6%*****	2.82E-22
Conditioning TB3	**70.8%*****	5.46E-5	**74.0%*****	5.69E-8	**81.3%*****	4.45E-6	**82.7%*****	3.66E-14
Conditioning TB4	**69.8%*****	1.32E-4	**73.2%*****	1.61E-7	**77.1%*****	9.44E-8	**77.2%*****	5.97E-10
Extinction TB1	**70.2%*****	1.11E-4	57.90%	0.090	**73.4%*****	6.34E-6	**60.3%***	0.026
Extinction TB2	54.30%	0.606	47.60%	0.656	**64.9%****	0.005	**60.3%***	0.026
Extinction TB3	59.60%	0.079	51.20%	0.789	54.30%	0.606	57.90%	0.090
Extinction TB4	51.00%	0.918	46.00%	0.423	50.00%	1.000	55.60%	0.247
Recall TB1 (CS+E vs. CS-)	**70.1%*****	2.24E-4	**70.2%*****	8.27E-6	**66.7%****	0.002	**71.8%*****	1.34E-6
Recall TB1 (CS+U vs. CS-)	**63.2%***	0.018	**70.2%*****	8.27E-6	**71.3%*****	9.06E-5	**80.6%*****	3.19E-12
Recall TB2 (CS+E vs. CS-)	55.20%	0.391	51.60%	0.788	58.60%	0.133	58.90%	0.059
Recall TB2 (CS+U vs. CS-)	47.10%	0.668	45.20%	0.323	59.80%	0.086	58.10%	0.088

Accuracies significantly higher than change level (two-sided binomial test, $p<0.05$) were bolded. TB: Trial-block; ***: $p<0.001$; **: $p<0.01$; *: $p<0.05$.

Supplementary Table 4. Basic information of external validation datasets 3-9.

Dataset	Training or testing	Demographic information	Key scanning information	Paradigm description	Original publications
Validation dataset 3	Testing	Sample size: 299 (58.6% female); Age (SD): 33.9 (10.1); Diagnosis: 299 HC	GE 3T MRI scanner, 8-channel head-coil. Functional data TR: 2400 ms, slice number: 47, voxel size: 3.0 × 3.0 × 3.0 mm.	Visual conditioning task, using two male three-dimensional virtual humanoid characters as CS, 16 trials per CS, 50% reinforcement rate. Stimulus duration: 6 s.	Vinberg et al. 2022[10].
Validation dataset 4	Testing	Sample size: 94 (69.4%); Age: 22.1 (3.3); Diagnosis: 94 HC	Philips 3T MRI scanner, 8- or 32-channel head-coil. Functional data TR: 2000 ms, slice number: 38 or 39, voxel size: 2.4 × 2.4 × 3.1 mm, or 3 × 3 × 3.3 mm.	Visual conditioning task, using face and house images as CS, 13 trials per CS, 46.2% reinforcement rate. Stimulus duration: 6 s.	Visser et al. 2021[11].
Validation dataset 5	Testing	Sample size: 48 (66.7%); Age: 23.5; Diagnosis: 24 PTSD, 24 TENC	Siemens 3T MRI scanner, 32-channel head-coil. Functional data TR: 2000 ms, slice number: 48, voxel size: 3 × 3 × 3 mm.	Visual conditioning task, using two categories of objects as CS, 24 trials per CS, 50% reinforcement rate. Stimulus duration: 4.5 ± 0.5 s.	Hennings et al. 2021[12].
Validation dataset 6	Testing	Sample size: 68 (66.2%); Age: 29.6 (15.9); Diagnosis: 68 HC	Siemens 3T MRI scanner, 32-channel head-coil. Functional data TR: 2000 ms, slice number: 39, voxel size: 3 × 3 × 3 mm.	Auditory conditioning task, using two tones as CS, 16 trials per CS, 50% reinforcement rate. Stimulus duration: 4 s.	Reddan et al. 2018[13].
Validation dataset 7	Testing	Sample size: 65 (50.7%); Age: 21.5 (2.1); Diagnosis: 65 HC	GE 3T MRI scanner. Functional data TR: 2000 ms, slice number: 36, voxel size: 3.125 × 3.125 × 3.8 mm.	Task assessed ‘subjective fear’ to intrinsically salient images. Participants were presented with 80 different pictures and were instructed to report the fearful state they experienced for the stimuli. Stimulus duration: 15 s.	Zhou et al. 2021[14].
Validation dataset 8	Testing	Sample size: 182 (52.0%); Age: 42.8 (7.3); Diagnosis: 182 HC	Siemens 3T MRI scanner, 12-channel head-coil. Functional data TR: 3000 ms, slice number: 34, voxel size: 3.125 × 3.125 × 3 mm.	Task examined negative affect induced by intrinsically salient images. Participants were presented with 15 neutral and 15 negative pictures and were instructed to report their emotional state. Stimulus duration: 7 s.	Chang et al. 2015[15].
Validation dataset 9	testing	Sample size: 59 (51.7%); Age: 20.8 (3.0); Diagnosis: 59 HC	Siemens 3T MRI scanner, 12-channel head-coil. Functional data TR: 2000 ms, slice number: 33, voxel size: 3.75 × 3.75 × 3 mm.	Tasks examined perception of physical pain or social pain. Participants were presented with heat/warm thermal stimuli or images of ex-partner/close friend. Stimulus duration: 15 s.	Woo et al. 2014[16].

PTSD: Post-traumatic stress disorder; TENC: Trauma-exposed non-PTSD control; HC: Healthy control; SD: Standard deviation.

Supplementary Table 5. Classification performance on external validation datasets 3-9.

Model	Metric	Dataset 3	Dataset 4	Dataset 5	Dataset 6	Dataset 7	Dataset 8	Dataset 9 (Physical pain)	Dataset 9 (Social rejection)
Conditioning TB1	Accuracy	78.3%	69.1%	79.2%	51.4%	76.9%	75.3%	49.2%	50.8%
	P-value	2E-23	2.6E-4	6.2E-5	0.90	1.5E-5	5.3E-12	~=1.0	~=1.0
	95% CI	[73.6%, 82.6%]	[59.6%, 77.7%]	[64.6%, 89.6%]	[41.2%, 63.2%]	[66.2%, 86.2%]	[69.2%, 81.3%]	[37.2%, 62.7%]	[37.3%, 64.4%]
Conditioning TB2	Accuracy	91.0%	90.9%	87.5%	83.8%	81.5%	70.3%	71.2%	61.0%
	P-value	4E-52	1.2E-16	1.0E-7	1.3E-8	2.8E-7	4.1E-8	0.002	0.12
	95% CI	[87.6%, 94.3%]	[83.0%, 95.7%]	[79.2%, 95.8%]	[75.0%, 91.2%]	[70.8%, 89.3%]	[63.7%, 76.9%]	[59.3%, 83.1%]	[49.2%, 72.9%]
Conditioning TB3	Accuracy	88.6%	89.4%	85.4%	67.6%	73.8%	72.0%	50.8%	61.0%
	P-value	1.6E-45	1.0E-15	6.2E-7	0.005	1.5E-4	2.7E-9	~=1.0	0.12
	95% CI	[85.0%, 92.0%]	[81.9%, 94.7%]	[72.9%, 93.8%]	[55.9%, 79.4%]	[63.1%, 84.6%]	[64.8%, 78.0%]	[37.3%, 64.4%]	[49.2%, 72.9%]
Conditioning TB4	Accuracy	79.6%	88.3%	83.3%	61.7%	80.0%	50.5%	57.6	57.6%
	P-value	7.5E-26	8.0E-15	3.3E-6	0.068	1.2E-6	0.94	0.30	0.30
	95% CI	[75.2%, 83.9%]	[80.9%, 94.7%]	[72.9%, 93.8%]	[50.0%, 72.1%]	[69.2%, 89.2%]	[43.4%, 57.7%]	[44.1%, 69.5%]	[44.1%, 69.5%]

P-values were based on two-sided binomial test. CI: Confidence interval.

Supplementary References

1. Milad, M. R. *et al.* Neurobiological basis of failure to recall extinction memory in posttraumatic stress disorder. *Biol. Psychiatry* **66**, 1075–1082 (2009).
2. Zeidan, M. A. *et al.* Estradiol modulates medial prefrontal cortex and amygdala activity during fear extinction in women and female rats. *Biol. Psychiatry* **70**, 920–927 (2011).
3. Milad, M. R. *et al.* Recall of fear extinction in humans activates the ventromedial prefrontal cortex and hippocampus in concert. *Biol. Psychiatry* **62**, 446–454 (2007).
4. Milad, M. R. *et al.* Deficits in conditioned fear extinction in obsessive-compulsive disorder and neurobiological changes in the fear circuit. *JAMA Psychiatry* **70**, 608–618 (2013).
5. Holt, D. J., Coombs, G., Zeidan, M. A., Goff, D. C. & Milad, M. R. Failure of neural responses to safety cues in schizophrenia. *Arch. Gen. Psychiatry* **69**, 893–903 (2012).
6. Marin, M.-F. *et al.* Association of resting metabolism in the fear neural network with extinction recall activations and clinical measures in trauma-exposed individuals. *Am. J. Psychiatry* **173**, 930–938 (2016).
7. Marin, M.-F., Hammoud, M. Z., Klumpp, H., Simon, N. M. & Milad, M. R. Multimodal categorical and dimensional approaches to understanding threat conditioning and its extinction in individuals with anxiety disorders. *JAMA Psychiatry* **77**, 618–627 (2020).
8. Sevinc, G. *et al.* Strengthened hippocampal circuits underlie enhanced retrieval of extinguished fear memories following mindfulness training. *Biol. Psychiatry* **86**, 693–702 (2019).

9. Seo, J. *et al.* Associations of sleep measures with neural activations accompanying fear conditioning and extinction learning and memory in trauma-exposed individuals. *Sleep* (2021).

10. Vinberg, K., Rosén, J., Kastrati, G. & Ahs, F. Whole brain correlates of individual differences in skin conductance responses during discriminative fear conditioning to social cues. *eLife* **11**, e69686 (2022).

11. Visser, R. M., Bathelt, J., Scholte, H. S. & Kindt, M. Robust bold responses to faces but not to conditioned threat: challenging the amygdala's reputation in human fear and extinction learning. *J. Neurosci.* **41**, 10278–10292 (2021).

12. Hennings, A. C., McClay, M., Drew, M. R., Lewis-Peacock, J. A. & Dunsmoor, J. E. Neural reinstatement reveals divided organization of fear and extinction memories in the human brain. *Curr. Biol.* **32**, 304–314 (2022).

13. Reddan, M. C., Wager, T. D. & Schiller, D. Attenuating neural threat expression with imagination. *Neuron* **100**, 994–1005 (2018).

14. Zhou, F. *et al.* A distributed fMRI-based signature for the subjective experience of fear. *Nat. Commun.* **12**, 6643 (2021).

15. Chang, L. J., Gianaros, P. J., Manuck, S. B., Krishnan, A. & Wager, T. D. A sensitive and specific neural signature for picture-induced negative affect. *PLOS Biol.* **13**, e1002180 (2015).

16. Woo, C.-W. *et al.* Separate neural representations for physical pain and social rejection. *Nat. Commun.* **5**, 5380 (2014).
