# Supplementary material for: Distributed neural representations of conditioned threat in the human brain
Source: Nat Commun. 2024 Mar 12;15:2231. doi: 10.1038/s41467-024-46508-0 (PMC10933283; doi:10.1038/s41467-024-46508-0)
Supplement: Supplementary file 3 — Reporting Summary [file 41467_2024_46508_MOESM3_ESM.pdf]

nature portfolio

Corresponding author(s): Mohammed R. Milad

Last updated by author(s): Jan 29, 2024

Reporting Summary

Nature Portfolio wishes to improve the reproducibility of the work that we publish. This form provides structure for consistency and transparency in reporting. For further information on Nature Portfolio policies, see our Editorial Policies and the Editorial Policy Checklist.

Statistics

For all statistical analyses, confirm that the following items are present in the figure legend, table legend, main text, or Methods section.

n/a	Confirmed	
☐	☒	The exact sample size (n) for each experimental group/condition, given as a discrete number and unit of measurement
☐	☒	A statement on whether measurements were taken from distinct samples or whether the same sample was measured repeatedly
☐	☒	The statistical test(s) used AND whether they are one- or two-sided<br>*Only common tests should be described solely by name; describe more complex techniques in the Methods section.*
☐	☒	A description of all covariates tested
☐	☒	A description of any assumptions or corrections, such as tests of normality and adjustment for multiple comparisons
☐	☒	A full description of the statistical parameters including central tendency (e.g. means) or other basic estimates (e.g. regression coefficient) AND variation (e.g. standard deviation) or associated estimates of uncertainty (e.g. confidence intervals)
☐	☒	For null hypothesis testing, the test statistic (e.g. F, t, r) with confidence intervals, effect sizes, degrees of freedom and P value noted<br>*Give P values as exact values whenever suitable.*
☒	☐	For Bayesian analysis, information on the choice of priors and Markov chain Monte Carlo settings
☒	☐	For hierarchical and complex designs, identification of the appropriate level for tests and full reporting of outcomes
☐	☒	Estimates of effect sizes (e.g. Cohen's d, Pearson's r), indicating how they were calculated

Our web collection on statistics for biologists contains articles on many of the points above.

Software and code

Policy information about availability of computer code

Data collection	Presentation 16.0, E-Prime 3.0, and SuperLab 5.0 were used for stimulus presentation.
Data analysis	Preprocessing of functional images was performed using fMRIPrep 20.0.2. Brain activation estimation was performed using Nistats 0.0.1rc. Classification analysis was performed using scikit-learn 0.23.1 and nilearn 0.9.2. The codes and data to generate the main figures and results are available at https://github.com/zhenfu-wen01/threat-mvpa and in a Zenodo repository at https://doi.org/10.5281/zenodo.10452949.

For manuscripts utilizing custom algorithms or software that are central to the research but not yet described in published literature, software must be made available to editors and reviewers. We strongly encourage code deposition in a community repository (e.g. GitHub). See the Nature Portfolio guidelines for submitting code & software for further information.

Data

Policy information about availability of data

All manuscripts must include a data availability statement. This statement should provide the following information, where applicable:

- Accession codes, unique identifiers, or web links for publicly available datasets
- A description of any restrictions on data availability
- For clinical datasets or third party data, please ensure that the statement adheres to our policy

The validation dataset 2 is available at NIMH Data Archive through collection ID 2393 (https://nda.nih.gov/edit_collection.html?id=2393). The validation dataset 4 is available in OpenNeuro database with the following accession numbers: ds003550 (https://doi.org/10.18112/openneuro.ds003550.v1.0.1), ds003553 (https://

doi.org/10.18112/openneuro.ds003553.v1.0.0), and ds003554 (https://doi.org/10.18112/openneuro.ds003554.v1.0.0). The validation dataset 5 is available at https://doi.org/10.17605/OSF.IO/QEG83. The validation dataset 6 is available at https://doi.org/10.17605/OSF.IO/68YWZ. The validation dataset 7 is available at http://doi.org/10.6084/m9.figshare.13271102.v2. The validation dataset 8 is available at https://identifiers.org/neurovault.collection:503. The validation dataset 9 is available at https://github.com/cocoanlab/interpret_ml_neuroimaging. The discovery dataset (including data from published and unpublished studies), and the validation datasets 1 and 3, are available upon request due to the need to establish data sharing agreements. There are no restrictions to who the data can be made available to, and there are no restrictions for data use for research. Requests for discovery dataset should be directed to M.R.M (mohammed.r.milad@uth.tmc.edu). Requests for the validation dataset 1 should be directed to S.W.L. (slazar@mgh.harvard.edu). Requests for validation dataset 3 should be directed to F.A. (fredrik.ahs@miun.se). The regional masks, predictive patterns, and trained classifiers are available at https://github.com/zhenfu-wen01/threat-mvpa and in a Zenodo repository at https://doi.org/10.5281/zenodo.1045294982. Source data are provided with this paper.

Research involving human participants, their data, or biological material

Policy information about studies with human participants or human data. See also policy information about sex, gender (identity/presentation), and sexual orientation and race, ethnicity and racism.

Reporting on sex and gender	We report using biological sex as reported in previous publications. Sex difference was not considered in any of the analyses as that is not a primary interest or objective for this study.
Reporting on race, ethnicity, or other socially relevant groupings	Demographic information were reported in previous publications. These data were not used in this study.
Population characteristics	See above.
Recruitment	Participants were recruited via advertisements posted on online platforms and public transportation advertisements.
Ethics oversight	The institutional review board of the Massachusetts General Hospital approved the studies that included participants from the discovery dataset and the validation datasets 1-2. Studies of validation datasets 3-9 were approved by the Institutional Review Boards of the corresponding research sites as stated in previous publications. All participants gave informed consent.

Note that full information on the approval of the study protocol must also be provided in the manuscript.

Field-specific reporting

Please select the one below that is the best fit for your research. If you are not sure, read the appropriate sections before making your selection.

☒ Life sciences ☐ Behavioural & social sciences ☐ Ecological, evolutionary & environmental sciences

For a reference copy of the document with all sections, see nature.com/documents/nr-reporting-summary-flat.pdf

Life sciences study design

All studies must disclose on these points even when the disclosure is negative.

Sample size	Sample size (N=1465) was based on the availability of existing study data. The sample size is larger than most of the previous threat conditioning studies.
Data exclusions	Participants were included if their structural and functional images were available.
Replication	Classification performance was assessed using cross-validation in the discovery dataset and validated in 2 external datasets that used the same paradigm. The generalizability and specificity of classifier were further validated using 7 external datasets.
Randomization	No randomization was performed as this study does not include experimental groups.
Blinding	Blinding is not relevant in this study because it does not include experimental groups.

Reporting for specific materials, systems and methods

We require information from authors about some types of materials, experimental systems and methods used in many studies. Here, indicate whether each material, system or method listed is relevant to your study. If you are not sure if a list item applies to your research, read the appropriate section before selecting a response.

Materials & experimental systems

n/a	Involved in the study
☒	☐ Antibodies
☒	☐ Eukaryotic cell lines
☒	☐ Palaeontology and archaeology
☒	☐ Animals and other organisms
☒	☐ Clinical data
☒	☐ Dual use research of concern
☒	☐ Plants

Methods

n/a	Involved in the study
☒	☐ ChIP-seq
☒	☐ Flow cytometry
☐	☒ MRI-based neuroimaging

Magnetic resonance imaging

Experimental design

Design type

Task fMRI, event-related design.

Design specifications

Trial numbers and trial duration varies across datasets, they were reported in previous publications. For the discovery dataset, and external datasets 1-2, each phase contained 32 trials, the duration of each conditioned stimulus (CS) was 6 s, and the duration of the inter-trial-interval (ITI) was 15 s on average.

Behavioral performance measures

No behavioral measures were recorded during the fMRI runs.

Acquisition

Imaging type(s)

Structural and functional images.

Field strength

3T.

Sequence & imaging parameters

Data were collected on multiple scanners using different acquisition parameters, which were described in the Methods and Materials section and also reported in previous publications.

Area of acquisition

Whole brain.

Diffusion MRI ☐ Used ☒ Not used

Preprocessing

Preprocessing software

fMRIPrep 20.0.2.

Normalization

The T1-weighted (T1w) were corrected for intensity non-uniformity with N4BiasFieldCorrection (ANTs 2.3.3) and used as T1w-reference throughout the preprocessing. The T1w-reference was skull-stripped, segmented into cerebrospinal fluid, white-matter and gray-matter, and then spatially normalized into the Montreal Neurological Institute (MNI) space (MNI152NLin2009cAsym) through nonlinear registration with antsRegistration (ANTs 2.3.3). The functional images were head-motion corrected using mcflirt (FSL) and slice-timing corrected using 3dTshift (AFNI). The preprocessed functional images were then co-registered to the T1w-reference using flirt (FSL) with the boundary-based registration (nine degrees of freedom), and spatially normalized into the MNI152NLin2009cAsym space by applying the parameters obtained from T1w-reference spatial normalization. Normalized functional images were resampled to 2 × 2 × 2 mm voxel size using Lanczos interpolation (ANTs 2.3.3) and smoothed with a 6-mm full-width half-maximum (FWHM) Gaussian kernel.

Normalization template

MNI152NLin2009cAsym

Noise and artifact removal

Motion parameters, high-pass temporal filtering (128 s) terms, volume-censoring indicators, and polynomial drift were included in first-level models as regressors of no interest.

Volume censoring

Volumes with framewise displacement > 0.9 mm were indicated as outliers in the model.

Statistical modeling & inference

Model type and settings

Multivariate pattern analysis was conducted in this study. Classifiers were trained to discriminate threat vs. safe cues.

Effect(s) tested

Accuracies in classifying threat vs. safe cues.

Specify type of analysis: ☐ Whole brain ☐ ROI-based ☒ Both

Anatomical location(s)	The CMA and BLA masks were defined based on amygdala masks constructed by Shackman and colleagues. The aHPC and pHPC masks were defined using the Harvard-Oxford subcortical probabilistic atlas (50% probability threshold, aHPC, and pHPC were separated by Y = -21 mm in MNI space). The dAI, vAI, and PI masks were defined based on clustering analysis of resting-state functional connectivity (Deen et al., 2011). The vmPFC, dACC, and sgACC masks were created using Neurosynth (Yarkoni et al., 2011) with "conditioning" as the keyword. An 8 mm sphere was created for each of the following identified peak coordinates: vmPFC (MNIxyz = -2, 46, -10), dACC (MNIxyz = 0, 14, 28), and sgACC (MNIxyz = 0, 26, -12).

Statistic type for inference (See Eklund et al. 2016)	Voxel-based thresholding was used to identify voxels significantly contributed to the classification.
Correction	FDR correction.

Models & analysis

n/a	Involved in the study
☒	☐ Functional and/or effective connectivity
☒	☐ Graph analysis
☐	☒ Multivariate modeling or predictive analysis

Multivariate modeling and predictive analysis	We used logistic regression with L2-regularization (or ridge regression) from scikit-learn as the classifier to discriminate threat vs. safe cues. The hyper-parameter of the L2-regularization term was selected from 20 equally distributed values between 0.01 and 100. The optimal hyperparameter was selected based only on training data. The classification performance was assessed using a 5-fold cross-validation procedure on the discovery dataset, and further validated on a total of 9 validation datasets.
